# Developing a Public Health Quality Tool for Mobile Health Clinics to Assess and Improve Care

**DOI:** 10.3390/ijerph23020141

**Published:** 2026-01-23

**Authors:** Nancy E. Oriol, Josephina Lin, Jennifer Bennet, Darien DeLorenzo, Mary Kathryn Fallon, Delaney Gracy, Caterina Hill, Madge Vasquez, Anthony Vavasis, Mollie Williams, Peggy Honoré

**Affiliations:** 1 Program in Medical Education, Harvard Medical School, Boston, MA 02115, USA; 2 Department of Anesthesia, Beth Israel Deaconess Medical Center, Boston, MA 02215, USA; 3 Harvard T.H. Chan School of Public Health, Boston, MA 02115, USA; 4 Family Van/Mobile Health Map, Harvard Medical School, Boston, MA 02115, USA; 5 Mobile Healthcare Association, St. Louis, MO 63146, USA; 6 Mobile Health Care Authority, San Francisco, CA 94117, USA; 7 Children’s Health Fund, New York, NY 10115, USA; 8 New York Foundling, New York, NY 10011, USA; 9 Department of Global Health and Social Medicine, Harvard Medical School, Boston, MA 02115, USA; 10 1A Wellness Group, Wenham, MA 01984, USA; 11 St. David’s Foundation, Austin, TX 78701, USA; 12 Madge Vasquez Consulting LLC, Austin, TX 78704, USA; 13 Callen-Lorde Community Health Center, New York, NY 10011, USA; 14 New York Hotel Trades Council & Hotel Association Health Center, Brooklyn, NY 11217, USA; 15 Mission Mobile Medical, Kernersville, NC 27284, USA; 16 DHHS/OS/Office for the Assistant Secretary for Health, Washington, DC 20201, USA; 17 LSU Health Sciences Center of Public Health and Medicine, New Orleans, LA 70112, USA

**Keywords:** mobile health clinics, public health, public health quality, quality assessment, quality improvement, self-assessment tool, health equity, community health, underserved

## Abstract

**Highlights:**

**Public health relevance—How does this work relate to a public health issue?**

- It illustrates how to translate broad public health quality aims into practical, measurable strategies.
- It offers a model for applying quality assessment and quality improvement processes in the practice of public health.

**Public health significance—Why is this work of significance to public health?**

- It fills a major gap, with a straightforward, evidence-based quality improvement tool for public health programs to meaningfully participate in quality assessment.
- The PHQ Tool highlights the value mobile clinics add to strengthening the broader public health and healthcare systems.

**Public health implications—What are the key implications or messages for practitioners, policy makers and/or researchers in public health?**

- Measuring quality in public health enables more targeted, effective population level improvements, giving practitioners clear, actionable strategies to enhance equity and impact.
- Aggregated quality data strengthens advocacy and informs funding and policy decisions, elevating mobile clinics as essential contributors to population health.

**Abstract:**

This report describes the development and deployment of the Public Health Quality Tool (PHQTool), an online resource designed to help mobile health clinics (MHCs) assess and improve the quality of their public health services. MHCs provide essential clinical and public health services to underserved populations but have historically lacked tools to assess and improve the quality of their work. To address this gap, the PHQTool was developed as an online, evidence-based, self-assessment resource for MHCs, hosted on the Mobile Health Map (MHMap) platform. This report documents the collaborative development process of the PHQTool and presents preliminary evaluation findings related to usability and relevance among mobile health clinics. Drawing from national public health frameworks and Honore et al.’s established public health quality aims, the PHQTool focuses on six aims most relevant to mobile care: Equitable, Health Promoting, Proactive, Transparent, Effective, and Efficient. Selection of the six quality aims was guided by explicit criteria developed through pilot testing and stakeholder feedback. The six aims were those that could be directly implemented through mobile clinic practices and were feasible to assess within diverse mobile clinic contexts. The remaining three aims (“population-centered,” “risk-reducing,” and “vigilant”) were determined to be less directly actionable at the program level or required system-wide or data infrastructure beyond the scope of individual mobile clinics. Development included expert consultation, pilot testing, and iterative refinement informed by user feedback. The tool allows clinics to evaluate practices, identify improvement goals, and track progress over time. Since implementation, 82 MHCs representing diverse organizational types have used the PHQTool, reporting high usability and identifying common improvement areas such as outreach, efficiency, and equity-driven service delivery. Across pilot and post-pilot implementation phases, a majority of respondents agreed or strongly agreed that the tool was user-friendly, relevant to their work, and appropriately scoped for mobile clinic practice. Usability and acceptance were assessed using descriptive statistics, including percentage agreement across Likert-scale items as well as qualitative feedback collected during structured debriefs. Reported findings reflect self-reported perceptions of feasibility, clarity, and relevance rather than inferential statistical comparisons. The PHQTool facilitates systematic quality assessment within the mobile clinic sector and supports consistent documentation of public health efforts. By providing a standardized, accessible framework for evaluation, it contributes to broader efforts to strengthen evidence-based quality improvement and promote accountability in MHCs.

## 1. Introduction

Mobile health clinics (MHCs) represent some of the United States’ most adaptable safety-net providers, delivering care across urban neighborhoods, rural and frontier towns, and post-disaster areas. Approximately 3000 MHCs operate nationwide, providing preventive care, primary care, health education, screenings, and connections to social services for populations often excluded from traditional health systems, including individuals experiencing homelessness, uninsured families, migrants, and rural residents [1].

Despite their reach, MHCs historically lacked standardized mechanisms to measure and improve public health impact. Unlike hospital systems that adopted formal quality improvement (QI) frameworks [2], MHCs operated with limited access to data systems or QI resources suited to their mobile, community-embedded context. The absence of a tailored framework impeded systematic evaluation and recognition of their contributions to population health. The Public Health Quality Tool (PHQTool) was developed to meet this need, offering a sector-specific, practical mechanism to assess and enhance quality performance. While recent research has highlighted the expanding role of mobile health clinics in advancing health equity, responding to public health emergencies, and delivering care in underserved settings, evaluations remain largely program-specific and would benefit from public health-oriented tools that allow mobile clinics across diverse contexts to assess service quality, implementation processes, and alignment with public health principles [3,4].

While the PHQTool was developed in the context of the United States mobile health clinic sector, mobile and outreach health services operate globally. The PHQTool was intentionally designed as an open-access, adaptable self-assessment resource that can be utilized by any mobile health program in a range of geographic and health system settings, including outside the United States. To date, the authors are not aware of comparable, sector-wide public health-oriented or quality-improvement tools designed explicitly for mobile clinics.

## 2. Methods

### 2.1. Public Health Quality Tool Development

#### 2.1.1. Early Foundations: The Family Van and Mobile Health Map

The development of the Public Health Quality Tool built upon prior work by The Family Van, a Boston-based mobile clinic launched in 1992 by Beth Israel Hospital and later affiliated with Harvard Medical School [5,6,7]. Recognizing the need for evidence to demonstrate value, The Family Van partnered in 2007 with the Mobile Health Clinics Association (MHCA) to create an evaluation framework quantifying return on investment (ROI). The resulting ROI calculator, published in 2009 [8], demonstrated cost avoidance associated with preventive care and catalyzed the formation of MobileHealthMap.org (MHMap), an online collaborative research platform for MHCs [9].

#### 2.1.2. National Partnerships and Public Health Quality Aims

In 2011, Honoré et al. (2011) introduced nine aims for public health quality: population-centered, equitable, proactive, health-promoting, risk-reducing, vigilant, transparent, effective, and efficient [10]. Recognizing MHMap’s ability to operationalize complex frameworks for frontline programs, Peggy Honoré, Director of Public Health Systems, Finance and Quality Programs in the U.S. Department of Health and Human Services (HHS) invited the MHMap team to adapt these aims for MHCs. While the U.S. Department of Health and Human Services’ framework has been widely cited in public health systems research, the specific process of adapting the nine public health quality aims into a practice-oriented set tailored for mobile clinics has not previously been documented in a peer-reviewed publication [11]. The adaptation described in this paper was informed by collaborative working sessions with MHCs, internal reports, and convenings and is presented here to increase transparency and reproducibility.

#### 2.1.3. Collaborative Design with Diverse Clinics

The mobile clinic sector, like the public health sector more broadly, has a need for practical tools that enable teams to communicate strengths to partners as well as identify opportunities for improvement. To ensure the PHQTool met the needs of MHCs, it was developed using an implementation science framework and an iterative, user-centered design process.

A collaborative working group of five mobile clinic programs—including the Casey Eye Institute at Oregon Health & Science University (OR), The Health Hut (LA), Maine Migrant Health Program (ME), St. David’s Dental Program (TX), and the Children’s Health Fund’s South Arizona Children’s Health Project (AZ)––was convened.

In addition to these partner clinics, advisors from established mobile and community-based health programs including The Family Van (MA) and Callen-Lorde Community Health Center (NYC) contributed to the collaborative design and refinement of the PHQTool by mapping routine MHC activities to the nine public health quality aims, convening meetings, facilitating discussions, and providing expert input based on prior experience with mobile health delivery and quality improvement. Following the initial conceptual mapping, the five mobile health clinics that had not participated in the tool’s development were invited to pilot test the prototype PHQTool. These clinics served as independent testers, providing structured feedback. Through iterative testing, six aims were identified as most actionable for the mobile clinic environment. Field testing and expert review ensured clarity, feasibility, and contextual relevance.

Daily MHC activities were mapped, such as providing linguistically appropriate health materials, real-time needs assessment, or adjusting routes to emerging community trends, to each public health quality aim. Iterative discussion revealed that while the original nine aims were visionary, three (“population-centered,” “risk-reducing,” “vigilant”) required infrastructure not available for many MHCs. The final version focused on six core, actionable aims: Equitable, Health Promoting, Proactive, Transparent, Effective, Efficient (Table 1). Clinics reported back on ambiguous language, gaps, and what “fit” or did not fit with street-based care realities. In addition to input from the five mobile clinics, the design team worked closely with Office of the Assistant Secretary of Health, advisors from the Institute of Healthcare Improvement, the National Quality Measures Clearinghouse and John Snow, Inc.

User feedback was collected at multiple stages of the PHQTool development through pilot use, structured discussions during regular meetings, and written feedback from participating mobile clinics. Participating clinics completed the assessment and engaged in structured debriefing sessions to evaluate feasibility, usability, clarity, actionability, and relevance to mobile clinic operations. Following each feedback cycle, the project team reviewed input to identify items that were unclear or not aligned with mobile clinic practices. Each revised version of the PHQTool was then tested with additional mobile clinic partners, as well as with funders and subject-matter experts, following the same feedback and revision process. Mobile clinic partners and external experts thus played a formative role in shaping the tool to ensure its applicability to public health practice. In total, six iterative revisions were completed prior to finalizing the PHQTool.

Debriefing with partner mobile clinics systematically addressed three domains. First, participants assessed whether the tool achieved its intended aims. Second, clinics evaluated feasibility, actionability, usability, clarity, reliability, priority for practitioners, and potential unintended consequences. Third, tool validity was assessed across two dimensions: (a) construct validity, evaluated by confirming that included strategies reflected established public health best practices and evidence where available; and (b) content validity, assessed through consultation with experts from the Institute for Healthcare Improvement, John Snow, Inc., the National Clearinghouse for Quality Measures, the Office for the Assistant Secretary for Health, the University of North Carolina, and the Institute for Community Health.

#### 2.1.4. Online Tool Construction and Release

The tool was implemented as a user-friendly, web-based resource modeled on the ROI calculator [8]. It allows users to complete self-assessments in under 30 min, identify strengths and gaps, and set one-year improvement goals. Open access and plain-language design ensured inclusivity for small and resource-limited organizations.

### 2.2. Pilot Testing and Iterative Refinement

#### 2.2.1. Early Pilots and Feedback

Initial pilot testing in 2014 involved 21 clinics, followed by a 2015 expansion to 45 clinics that completed the tool during the 2015 implementation period [11]. The 2015 implementation data are included to document the original development, refinement, and early field testing of the PHQTool. These findings provide a historical baseline demonstrating feasibility, clarity, and perceived relevance when the tool was first disseminated for broader use. Feedback guided language refinement and tool structure to better reflect real-world operations. Partner mobile clinics were selected with convenience sampling. Selection prioritized heterogeneity across key dimensions of the mobile clinic sector, including geographic setting, populations served, clinical focus, organization structure, and funding models. Clinics were invited to participate based on their willingness and capacity to engage in iterative testing and feedback, which were essential for tool development.

#### 2.2.2. Sector Engagement and Dissemination

Adoption of the PHQTool was advanced through presentations at major public health forums, including the American Public Health Association, the National Healthcare for the Homeless Council, the Institute for Healthcare Improvement forums, and through webinars hosted by the Health Resources and Services Administration (HRSA) and the Federal Office of Rural Health Policy (FORHP). Federal partners facilitated introductions to large-scale grantees, and the tool soon gained traction with hospital-linked programs, rural health departments, and philanthropic funders seeking ROI and quality documentation. The tool’s voluntary, anonymous and supportive framework encouraged engagement without the perception of external oversight.

## 3. Results

### 3.1. Application and Quality Priorities

During the 2015 implementation phase, approximately 45 mobile health clinics completed the PHQTool. Among the 22 respondents to the second implementation round of evaluation items,

- 96% rated usability as high;
- ≥70% found questions relevant to their practice;
- 83% planned to strengthen work in at least one quality aim.

More than 60% used the tool to inform annual planning, grant reporting, or performance evaluation. The most common improvement goals involved proactive needs assessment, cost-tracking for efficiency, and equity-focused outreach. The three most frequently selected goals were:

- Proactive aim**:** Increase capacity for real-time needs analysis and client feedback systems.
- Efficient aim: Expand cost tracking and develop simple ROI calculations for funders.
- Equitable aim: Improve service location convenience and offer trusted formats for health education in multiple languages.

### 3.2. Uptake and Clinic Characteristics

Since the post-COVID-19 revision, 82 MHCs have used the PHQTool. Participating programs include Federally Qualified Health Centers (FQHCs), hospital affiliates, nonprofit agencies, and university-based clinics operating in urban, suburban, and rural settings, including disaster-response deployments.

The sample includes:

- Organizations with federal (40%), philanthropic (65%), public (56%), and private (67%) funding (many reporting multiple sources).
- Urban, suburban, and rural catchment areas, including post-disaster deployments after hurricanes and during the COVID-19 pandemic.

## 4. Discussion

The co-developed PHQTool demonstrates that mobility and community orientation can coexist with rigorous quality improvement. By simplifying evaluation processes and centering equity, MHCs can participate fully in quality culture without extensive infrastructure.

Key lessons include:

- Accessible quality improvement encourages sustained self-evaluation across diverse organizations.
- Equity integration ensures that assessment frameworks reflect community realities.
- Data combined with narrative context strengthens advocacy with funders and health systems.

As health systems expand out-of-facility care to address inequities [12,13], tools such as this provide a tested mechanism for structured evaluation. The COVID-19 pandemic underscored the importance of agile, data-driven mobile responses [14,15,16]; the PHQTool supports readiness and accountability for such efforts.

The PHQTool was intentionally developed as a resource that strengthens and standardizes care delivery processes that underlie high quality public health practice. Improvements with the PHQTool reflect intentional changes in implementation practices and processes, such as with data sharing, outreach strategies, and community engagement. This process-oriented approach is consistent with public health quality assessment and is particularly appropriate for mobile clinics, which operate across diverse settings with diverse data measurement and evaluation capacities.

Future work will focus on iterative refinement of the PHQTool to reflect evolving mobile care models and public health priorities; expansion of the tool to additional mobile settings; and exploration of how improved implementation practices relate to measurable changes in outcomes. In addition, qualitative inquiry into how clinics use the PHQTool for planning, funding, and internal quality improvement will further inform refinement. Additional research with the PHQTool can assess measurable improvements in outcomes over time. Potential outcome domains aligned with the six public health quality aims include client trust and satisfaction (equitable), improvements in preventive indicators and biometrics (health promoting), strengthened community relationships and responsiveness (proactive), increased stakeholder engagement and data sharing (transparent), and program sustainability or efficiency gains (effective and efficient). The PHQTool offers a roadmap for longitudinal evaluation, in which process improvements serve as a foundation for public health outcomes.

This report has a few limitations that should be considered. Usability and acceptance data presented here derive from the 2015 implementation period and are included primarily to document the timeline, iterative refinement process, and early field testing of the PHQTool. These findings provide historical context for how the tool was initially operationalized, tested, and refined (Table 2). During and after the COVID-19 pandemic, the PHQTool and its hosting platform underwent structural and technical upgrades that limited the continuity of comparable data across time. Participation in pilot testing and implementation was also voluntary, introducing potential self-selection bias towards clinics with more interest in quality improvement.

Findings should also be situation within this report’s implementation-focused design. Findings related to usability, acceptance, and relevance findings were assessed using descriptive metrics and qualitative input to support iterative improvements rather than inferential statistical analysis. This approach aligns with implementation processes for early phase tool development prioritizing feasibility and usability. Future research could build on this work through larger-scale validation studies, including formal reliability testing and quantitative analyses examining associations between PHQTool use and selected outcome measures.

## 5. Conclusions

Mobile health clinics have long advanced equity and effectiveness through direct community engagement. The PHQTool provides a scalable, evidence-based mechanism to document and improve these contributions. Embedding continuous quality improvement within mobile health operations is both feasible and necessary. MHMap and the PHQTool offer a replicable framework for equity-oriented evaluation across population-based health initiatives.

## Figures and Tables

**Table 1:** Public Health Quality Tool Aims and Sample Strategies (see Table A1).

Public Health Aim	Example Strategies
Equitable	Affordable services for uninsured/underinsured clients; Materials at <6th-grade literacy; 2+ languages offered; Staff who reflect community diversity
Health Promoting	Health education/counseling; Evidence-based clinical interventions (e.g., vaccines); Addressing contextual and social determinants
Proactive	Analyzing community health data; Regular client surveys; Adaptive service models for emerging needs; Staff emergency training
Transparent	Public reporting of program/process/outcome data; Accessible governance and finance information
Effective	Prioritizing evidence-based interventions; Assessing changes in knowledge/behavior/health outcomes post-intervention
Efficient	Tracking costs/ROI per person served, streamlining operations without sacrificing access or quality

**Table 2:** Historical Timeline: Key Moments in Mobile Health Map and Quality Tool Innovation.

Year	Milestone
1992	The Family Van launches in Boston
2007	The Family Van, Mobile Healthcare Association (MHA), Harvard School of Public Health and health economist Paul Cote Jr., MBA begin Return on Investment (ROI) pilot
2009	The Family Van, MHA, The Boston Children’s Hospital Department of Information Technology and Harvard Medical School (HMS) Department of Bioinformatics launch Mobile Health Map.org (MHM) and the online ROI calculator
2011	MHM team presents ROI calculator to Health Resources Service Administration (HRSA) and Federal Office of Rural Health Policy (FORHP) in Washington, DC Honoré et al. [10] publishes public health quality aims
2012	Health and Human Services (HHS) and Office of Minority Health (OMH) sponsor a convening inviting over 100 Representatives of Agencies and Offices including Peggy A. Honoré, Director of Public Health Systems, Finance, and Quality Program, Office of Healthcare Quality/Office of the Assistant Secretary for Health, Department of Health and Human Services
2012	Honore invited and fund MHMap to convert the Public Health Quality Aims (PHQA) to concrete Metrics and create an online tool for PHQ
2012–2013	Collaborative working group with five flagship clinics create quality assessment tool
2014	Public Health Quality Tool (PHQTool) launches online, free for any Mobile clinic registered on MHMap, first public presentation American Public Health Association (APHA) annual meeting New Orleans
2015	Version 2 released after user feedback
2016–2019	National dissemination of PHQTool with invited presentations at APHA annual meetings, Institute for Health Improvement (IHI) forum, National Quality Partners, MHA Annual Conferences, HRSA, FOPHP, Agency for Healthcare Research and Quality (AHRQ), Weitzman Institute, and an interview on National Public Radio
2020	The Leon Lowenstein Family Foundation funds MHMap, and helps scale MHCs during COVID Response, MHMap and Quality tool rebuilt
2023–2025	82+ clinics nationwide complete quality tool; mobile clinics nationwide use MHMap tools for advocacy

## Data Availability

The original contributions presented in this study are included in the article. Further inquiries can be directed to the corresponding author.

## Abbreviations

The following abbreviations are used in this manuscript:

PHQTool	Public Health Quality Tool
MHCs	Mobile Health Clinics
MHMap	Mobile Health Map
QI	Quality Improvement
MHCA	Mobile Health Clinics Association
ROI	Return on Investment
HHS	Health and Human Services
HRSA	Health Resources and Services Administration
FORHP	Federal Office of Rural Health
FQHC	Federally Qualified Health Center

## Appendix A

**Table A1:** Public Health Quality Tool, Definitions and Metrics.

Public Health Quality Tool Metrics	Definition	Metrics
Equitable	Does your program advance health equity? An equitable program works towards health equity by addressing health disparities—the gaps in quality of health or health care due ot the social determinants of health (like race or ethnicity, education level, or socioeconomic status).	i. Services will be affordable for those who are uninsured, underinsured, or low-income Use strategy now Use more next year Don’t use and don’t plan to implement within next year ii. Written information will be easy to understand for those with low-literacy or language barriers Use strategy now Use more next year Don’t use and don’t plan to implement within next year iii. Locations will be convenient Use strategy now Use more next year Don’t use and don’t plan to implement within next year iv. The staff will speak the 2 most common languages Use strategy now Use more next year Don’t use and don’t plan to implement within next year v. The staff will reflect the diversity of the population served Use strategy now Use more next year Don’t use and don’t plan to implement within next year
Health Promoting	Is your program health promoting? A health promoting program adopts policies and strategies that advance safe practices by providers and the population and that increase the probability of positive health behavior and outcomes.	i. Offer counseling and education Use strategy now Use more next year Don’t use and don’t plan to implement within next year ii. Use clinical interventions Use strategy now Use more next year Don’t use and don’t plan to implement within next year iii. Implement long-lasting clinical interventions such as vaccinations Use strategy now Use more next year Don’t use and don’t plan to implement within next year iv. Change the context to people’s health, e.g., by offering healthy food in schools Use strategy now Use more next year Don’t use and don’t plan to implement within next year v. Addresses the social determinants of health including poverty and discrimination Use strategy now Use more next year Don’t use and don’t plan to implement within next year
Proactive	Is your program proactive? Proactive programs adopt policies and sustainable practices in a timely manner, while mobilizing rapidly to address new and emerging threats and vulnerabilities.	i. Analyze community health reports and community data Use strategy now Use more next year Don’t use and don’t plan to implement within next year ii. Get feedback from people you serve on a regular basis Use strategy now Use more next year Don’t use and don’t plan to implement within next year iii. Review program data for emerging needs among your target population Use strategy now Use more next year Don’t use and don’t plan to implement within next year iv. Adjust services to address emerging needs Use strategy now Use more next year Don’t use and don’t plan to implement within next year v. Train personnel in emergency response Use strategy now Use more next year Don’t use and don’t plan to implement within next year
Transparent	How transparent is your program? Transparency ensures openness in the delivery and practices, with particular emphasis on valid, reliable, accessible, timely, and meaningful data that are readily available to stakeholders, including the public.	i. Operational data Share data now Share next year Don’t share and don’t plan to share within next year ii. Equity data Share data now Share next year Don’t share and don’t plan to share within next year iii. Outcomes data Share data now Share next year Don’t share and don’t plan to share within next year iv. Governance data Share data now Share next year Don’t share and don’t plan to share within next year v. Financial data Share data now Share next year Don’t share and don’t plan to share within next year
Effective & Efficient	How effective and efficient is your program? Effective and efficient programs use evidence, science, and best practices to achieve optimal results in areas of greatest need. Understands costs and benefits of public health interventions, to facilitate the optimal use of resources to achieve desired outcomes.	i. Use evidence-based interventions (programs proven to be effective and efficient) Use strategy now Use more next year Don’t use and don’t plan to implement within next year ii. Measure changes in knowledge, behavior, or health after intervention Use strategy now Use more next year Don’t use and don’t plan to implement within next year iii. Measure differences in a group’s health when compared to another group that haven’t received your program Use strategy now Use more next year Don’t use and don’t plan to implement within next year iv. Track expenses per individual served Use strategy now Use more next year Don’t use and don’t plan to implement within next year v. Track return on investment Use strategy now Use more next year Don’t use and don’t plan to implement within next year
